# 驱动基因阴性非小细胞肺癌脑转移免疫治疗的研究进展

**DOI:** 10.3779/j.issn.1009-3419.2018.08.06

**Published:** 2018-08-20

**Authors:** 爽 张, 菁菁 柳, 长良 杨

**Affiliations:** 130012 长春，吉林省肿瘤医院胸部肿瘤内科 Department of Thoracic Oncology, Jilin Provincial Cancer Hospital, Changchun 130012, China

**Keywords:** 肺肿瘤, 脑转移, 免疫检查点抑制剂, 驱动基因阴性, Lung neoplasms, Brain metastases, Immune checkpoint inhibitor, Driver gene negative

## Abstract

脑转移是非小细胞肺癌（non-small cell lung cancer, NSCLC）常见的转移部位和导致死亡的主要原因。酪氨酸激酶抑制剂（tyrosine kinase inhibitor, TKI）改善了驱动基因阳性NSCLC患者的生存，同时也为驱动基因阳性NSCLC脑转移患者带来了福音，然而对于驱动基因阴性脑转移NSCLC仍然缺少有效的治疗手段。近年来，免疫靶向治疗取得突破性进展，成为晚期NSCLC，尤其是驱动基因阴性患者一、二线治疗选择。免疫靶向治疗在NSCLC的特殊人群——脑转移患者尤其是驱动基因阴性患者中发挥怎样的作用已经成为研究者关注的焦点。本文将总结免疫靶向治疗在NSCLC脑转移尤其是驱动基因阴性患者中的研究进展，分析现有研究的局限和未来面临的挑战。

## Abstract (en)

Brain metastasis was a common metastasis site and leading cause of death in non-small cell lung cancer (NSCLC). Tyrosine kinase inhibitors had improved survival of NSCLC patients with positive drive gene. It also brings good news to NSCLC patients with positive drive gene and brain metastases. However, there is still no effective treatment for NSCLC patients with drive gene-negative and brain metastases. In recent years, immunotherapy has made breakthrough progress and become important first and second line treatment options of NSCLC especially in patients with drive gene-negative. The role of immunotherapy in specific populations of NSCLC-brain metastasis patients, especially drive gene-negative patients has become the focus of attention. In this report, we review the research progress of immunotherapy in NSCLC with brain metastases, especially in driver-negative patients, analyze the limitations of existing research and future challenge.

## 1 前言

分子靶向药物使驱动基因阳性非小细胞肺癌（non-small cell lung cancer, NSCLC）患者取得生存获益的同时，也为脑转移患者带来希望。然而驱动基因阴性NSCLC脑转移的治疗一直是困扰研究者和临床医生的难题。与驱动基因阳性NSCLC脑转移不同，驱动基因阴性NSCLC发生脑转移的比例略低，占20%左右^[1, 2]^，累计脑转移发生率在28%-38%^[3, 4]^，且更常见于单发或者较少数目的脑转移^[1, 5]^。目前，局部放疗仍然是驱动基因阴性NSCLC脑转移的标准治疗选择，化疗、贝伐珠单抗也是重要的治疗手段，但是疗效有限，生存期不足8个月^[1, 3-5]^，而且耐受性差。因此，迫切需要新的疗法改善驱动基因阴性NSCLC脑转移患者的治疗现状。

免疫治疗在NSCLC领域取得了突破性进展，从最初的Nivolumab、Pembrolizumab、Atezolizumab获批用于NSCLC二线治疗，到Pembrolizumab单药、Pembrolizumab联合化疗一线治疗无*EGFR*突变和*ALK*融合突变晚期NSCLC，再到Durvalumab用于放化疗后Ⅲ期NSCLC的维持治疗，免疫靶向治疗已经成为NSCLC治疗中不可或缺的部分。然而，免疫靶向药物在特殊人群——驱动基因阴性NSCLC脑转移患者中发挥的作用仍然不明确。

## 2 免疫靶向药物单药治疗NSCLC脑转移患者

在NSCLC免疫靶向治疗的临床研究中，虽然多数并未排除脑转移患者，但对纳入的脑转移患者进行了严格的限定，要求无需使用激素治疗，大部分研究排除有症状脑转移和癌性脑膜炎的患者。已经发表的NSCLC免疫靶向治疗的临床研究中，脑转移患者入组比例较低，仅占10%左右^[6-13]^。目前，免疫靶向治疗脑转移的结果主要来自临床研究亚组分析及回顾性研究结果，除了KEYNOTE 024研究为NSCLC一线治疗且纳入驱动基因阴性患者外，其余的研究均为二线及以上治疗的患者，研究纳入部分TKI治疗进展的驱动基因阳性NSCLC，这些研究中是以高加索裔为主，突变人群比例低，研究中对脑转移患者是否存在驱动突变未进行严格区分。

OAK研究^[12]^是一项Atezolizumab对比多西他赛治疗经治晚期NSCLC的3期临床研究。Atezolizumab组和多西他赛组分别纳入38例和47例无症状、既往进行局部治疗后稳定的脑转移患者。与多西他赛治疗相比，Atezolizumab治疗脑转移患者可以降低39%的疾病进展风险，减少45%的死亡风险^[14]^。对于无症状、既往接受局部治疗稳定的脑转移患者，Atezolizumab治疗较化疗有显著的总生存（overall survival, OS）获益。OAK研究亚组结果支持进一步开展Atezolizumab治疗NSCLC脑转移患者的临床研究。

汇集CheckMate 017（3期研究）、CheckMate 057（3期研究）、CheckMate 063（2期研究）3项研究的Nivolumab治疗经治晚期NSCLC的汇总分析^[15]^中纳入971例患者，Nivolumab治疗组544例患者，脑转移患者46例，多西他赛治疗组427例，脑转移患者42例，对脑转移患者进行亚组分析发现，Nivolumab治疗组和多西他赛治疗组3个月出现中枢神经系统（central nervous system, CNS）新病灶的比例分别为7%和12%，6个月出现CNS新病灶的比例分别为13%和17%，中位OS分别为8.4个月和6.2个月。与化疗相比，Nivolumab单药治疗经治脑转移有改善OS和降低颅内新病灶发生的趋势。

KEYNOTE 024研究^[11]^是一项Pembrolizumab一线治疗PD-L1高表达驱动基因阴性晚期NSCLC的3期研究。研究中纳入CNS转移患者，Pembrolizumab组18例，化疗组10例。这部分患者中Pembrolizumab治疗组在无进展生存期（progression-free survival, PFS）和OS上均有获益趋势。

一些回顾性研究也分析了免疫靶向药物治疗NSCLC脑转移的疗效。目前的数据主要来自Nivolumab治疗的经验。一项来自以色列的研究^[16]^中纳入260例晚期NSCLC患者，其中CNS转移患者55例，CNS转移患者和无CNS转移患者的中位OS分别为7.0个月和5.2个月（*P*=0.5），可见脑转移NSCLC患者接受Nivolumab治疗的生存获益与无脑转移患者相似。意大利Nivolumab治疗的扩展应用计划（Expanded Access Programme, EAP）项目分别对晚期肺鳞癌^[17]^和晚期非鳞癌NSCLC^[18]^进行研究。肺鳞癌研究纳入371例经治的Ⅲb期/Ⅳ期患者，其中37例为无症状脑转移患者，Nivolumab治疗CNS患者客观缓解率（objective response rate, ORR）为19%，疾病控制率（disease control rate, DCR）为49%，1年的OS率为35%，1年PFS率为31%^[17]^。在Nivolumab治疗晚期非鳞癌NSCLC的研究中纳入1, 588例患者，其中409例无症状脑转移患者，研究发现CNS转移患者的ORR为17%，DCR为40%，中位OS为8.6个月，1年的OS率为43%^[18]^。这两项研究中Nivolumab治疗无症状脑转移NSCLC的疗效分别与Nivolumab治疗肺鳞癌和非鳞癌随机对照3期临床研究、CheckMate 017研究和Checkmate 057研究的结果相似，提示Nivolumab对颅内外病灶同样有效。

以上亚组分析或者回顾性分析，样本量有限，CNS转移，尤其是驱动基因阴性NSCLC能否从免疫靶向治疗中获益还需要大样本的、随机对照的临床研究进行评估。

目前专门针对NSCLC脑转移的前瞻性研究非常有限。FIR研究^[13]^探讨Atezolizumab治疗PD-L1表达的局部进展或转移性NSCLC的疗效，该研究包含3个队列，其中一个队列纳入≥2线，接受过治疗的脑转移患者，目前入组13例，ORR为23%，中位PFS和OS分别为4.3个月和6.3个月。CheckMate-012研究^[15]^同样有一个队列针对脑转移患者，纳入≥1个无症状CNS转移，未进行局部治疗的NSCLC接受Nivolumab，入组的12例患者中1例获得完全缓解（complete response, CR），1例获得部分缓解（partial remission, PR），ORR为16.7%，中位OS为8.0个月，中位PFS为1.6个月。以上研究结果提示对于NSCLC伴有无症状CNS转移的患者或许可从单药Nivolumab治疗中获益。

在Pembrolizumab治疗CNS转移的前瞻性2期研究^[19]^中纳入至少有1个未经治疗或进展的5 mm-20 mm的CNS转移灶，无中枢症状或者不需糖皮质激素治疗的PD-L1阳性的NSCLC，18例患者中脑转移的ORR为33%，总ORR为33%，这项研究初步证实Pembrolizumab对选择性NSCLC脑转移病灶有效，颅内病灶与颅外病灶的缓解率具有一致性。

从目前的研究结果看，对于存在CNS转移的NSCLC患者，免疫靶向单药已初步显示出临床获益，Atezolizumab的生存获益最为突出，PD-L1抑制剂和PD-1抑制剂对于CNS转移的疗效是否有差异还需要进一步探索。

## 3 免疫靶向治疗CNS转移的联合策略

局部放疗是脑转移的标准治疗方法，免疫治疗与放疗联合是否能够进一步提高疗效呢？研究发现放疗促进肿瘤和周围组织释放各种因子，导致肿瘤细胞发生免疫原性死亡，活化抗原提呈细胞，使各种细胞因子释放，激活T细胞，同时肿瘤细胞表达主要组织相容性复合物I（major histocompatibility complex type I, MHC I）、钙网蛋白等，促进免疫系统攻击肿瘤细胞^[20]^。

KEYNOTE-001研究^[10]^是Pembrolizumab单药治疗经治晚期NSCLC的1期研究，该研究对免疫治疗前接受放疗的单中心数据二次分析发现^[21]^，42例患者接受放疗，其中38例接受颅外放疗，4例接受颅内放疗，55例患者没有接受放疗，接受放疗患者与未接受放疗患者的中位PFS分别为4.4个月和2.1个月（*P*=0.019），中位OS分别为10.7个月和5.3个月（*P*=0.026），可见免疫治疗联合放疗可使晚期NSCLC患者获得更长的PFS和OS，免疫治疗联合放疗在NSCLC发挥协同作用。

一些回顾性研究分析了免疫治疗联合放疗在CNS转移的NSCLC中的疗效。一项研究^[22]^纳入17例接受免疫靶向治疗和立体定向放疗（stereotatic radiosurgery treatment, SRS）治疗的NSCLC，从放疗开始的OS为5.6个月，从诊断开始的OS为17.9个月，免疫治疗前或治疗中进行SRS的患者和免疫治疗后进行SRS的患者6个月颅内ORR分别为57%和0%，可见免疫治疗联合放疗，尤其免疫治疗前或同步接受SRS能够使CNS转移得到控制。另一项回顾性研究^[23]^分析了260例存在脑转移的实体瘤患者接受SRS的疗效，纳入NSCLC患者157例，黑色素瘤70例，肾癌33例，其中有30%（79例）的患者同时接受了免疫靶向治疗。研究发现仅接受SRS患者（181例），SRS同时联合免疫治疗患者（28例）和SRS非同步联合免疫治疗患者（51例）的中位OS分别为12.9个月、24.7个月和14.5个月，1年PFS率分别为82%、79%和88%，新出现脑转移的中位数目分别为4（范围：0-96）、4（范围：0-26）和2（范围：0-11），研究认为免疫治疗与SRS同步治疗能够改善CNS转移患者的OS，减少新的颅内病灶出现。可见免疫靶向治疗联合SRS，尤其是同步治疗具有更好的抗肿瘤活性，期待前瞻性研究进一步验证。

研究者在关注免疫靶向治疗联合放疗疗效的同时也关注是否增加了毒性。一项回顾性分析^[24]^对163例接受颅内放疗NSCLC CNS转移患者的安全性进行了评估，其中50例接受了免疫靶向治疗，与颅内放疗相比，联合治疗颅内放射毒性的发生率相似，提示CNS转移患者接受颅内放疗联合免疫治疗是安全可行的。但另一项回顾性研究^[25]^发现SRS联合免疫靶向治疗较SRS单独治疗CNS转移癌发生有症状放射性脑坏死的比例明显增高，分别为20%（23/115）和6.8%（25/365），研究提示免疫治疗联合SRS需要慎重选择。

回顾性研究中，免疫靶向治疗联合放疗在NSCLC脑转移患者初步看到协同作用，但仍在探索阶段，有3项前瞻性研究正在进行（表 1），除了需要进一步验证疗效外，免疫与放疗联合的最佳时序、联合治疗的剂量，相关毒性以及适合的人群也是未来需要解决的问题。

**1:** 正在进行的脑转移性NSCLC放射治疗和免疫治疗研究综述 Summary of ongoing studies combining radiation therapy and immunotherapy in NSCLC with brain metastases

ClinicalTrials.gov Identifier	Phase	Arms	Estimated enrollment
NCT02696993	1/2	Nivo+SRS *vs* Nivo+WBRT *vs* Nivo+Ipi+SRS *vs* Nivo+Ipi+WBRT	80
NCT02978404	2	Nivo+SRS	60 (NSCLC+Renal cell carcinoma)
NCT02858869	1	Pembro+SRS (SRS: 6 Gy *vs* 9 Gy *vs* 18 Gy-21 Gy)	30 (NSCLC+Melanoma)
NSCLC: non-small cell lung cancer; SRS: stereotatic radiosurgery treatment; WBRT: whole brain radiation therapy.			

免疫靶向治疗与其他药物的联合治疗CNS转移瘤也在探索中，包括免疫联合抗血管^[26]^、免疫与免疫联合^[27]^、免疫联合透过血脑屏障化疗药物的联合^[28]^治疗脑胶质瘤、黑素瘤的研究。目前还缺少免疫靶向治疗联合其他药物在NSCLC脑转移患者的数据，来自其他实体瘤的小样本研究的初步结果看到免疫靶向治疗联合其他药物对颅内病灶控制充满前景，值得在NSCLC CNS转移人群中进行进一步研究。目前免疫治疗联合贝伐珠单抗（NCT02681549）和双免疫治疗（checkmate819研究特殊人群队列）CNS转移NSCLC的前瞻性研究正在进行。

## 4 CNS转移免疫治疗优势人群筛选

免疫靶向治疗颅内转移瘤同样需要筛选适合的人群，寻找理想的标志物。一些研究已经发现PD-L1表达情况与免疫治疗疗效相关，PD-L1表达能否预测免疫治疗对CNS转移的疗效？在Pembrolizumab治疗存在脑转移的NSCLC和黑色素瘤的前瞻性2期研究^[19]^中，要求纳入的NSCLC患者最近一次系统治疗前任何部位的肿瘤组织PD-L1阳性（≥1%），研究发现颅内病灶的应答率与颅外病灶的应答率一致，是否提示颅外病灶PD-L1表达可作为筛选CNS转移患者接受免疫靶向治疗的标志物？然而现有的研究也提示，肺部原发灶与脑转移灶的免疫表型存在差异。一项纳入11例原发灶与脑转移灶配对标本的*EGFR*突变阴性的NSCLC患者的回顾性分析^[29]^发现，配对标本中PD-L1的表达不一致率为36.3%。梅奥诊所的一项纳入73例NSCLC原发灶和脑转移灶的分析^[30]^中发现不仅颅内外肿瘤细胞PD-L1表达不一致，颅内外肿瘤组织中侵润的淋巴细胞上PD-L1表达也存在差异。除了PD-L1表达外，研究者也探索了肿瘤侵润淋巴细胞、肿瘤突变负荷（tumor mutation burden, TMB）、新抗原负荷等免疫治疗相关标志物在原发灶与脑转移灶之间的异质性。一项回顾性研究^[31]^分析了20例原发灶与脑转移的配对标本，研究发现，与原发灶相比，脑转移灶侵润的T淋巴细胞克隆数更少，其中13对NSCLC原发灶与CNS转移灶配对标本DNA测序，TMB在CNS转移灶更高，而在新抗原负荷的分析了中发现原发灶与转移灶没有明显差异。可见，颅外病灶不能完全代替CNS转移灶进行患者接受免疫治疗的筛选，但是CNS转移灶肿瘤组织获取困难，需要寻找CNS转移灶免疫微环境理想的替代标本。脑脊液可能是探索颅内病灶免疫状态的窗口，但是目前缺少脑脊液中免疫标志物的研究，此外，外周血中特定的免疫细胞亚群或细胞因子能否成为脑转移患者接受免疫治疗疗效预测的标志物也是未来探索的方向。

## 5 总结

免疫靶向治疗在NSCLC治疗领域快速崛起，从目前小样本的亚组分析和回顾性研究中，已见到部分NSCLC脑转移患者能够从免疫靶向治疗中获益，专门针对NSCLC CNS转移免疫靶向治疗的前瞻性研究正在进行。免疫靶向治疗在NSCLC中的特殊人群—驱动基因阴性脑转移患者能否再下一城，续写新的神话值得期待。联合治疗是免疫治疗的发展趋势，在其他瘤种CNS转移治疗充满前景，NSCLC脑转移中免疫联合策略一样炙手可热。CNS转移免疫靶向治疗优势人群筛选势在必行，理想的标志物是什么，脑脊液、外周血能否代替脑转移肿瘤组织用于标志物筛选等诸多问题有待探索。
